# Epalrestat improves motor symptoms by reducing oxidative stress and inflammation in the reserpine induced mouse model of Parkinson’s disease

**DOI:** 10.1002/ame2.12097

**Published:** 2019-12-30

**Authors:** Md. Mahbubur Rahman, Rupali Rani Chakraborti, Md. Abdullah Potol, Ariful Haque Abir, Ozayra Sharmin, Mahabub Alam, Md. Fazlur Rahman Khan, Rownock Afrin, Humayra Jannat, Rasiqh Wadud, Zaki Farhad Habib

**Affiliations:** ^1^ Laboratory of Pharmacology Department of Pharmaceutical Sciences School of Health & Life Sciences North South University Dhaka Bangladesh

**Keywords:** bradykinesia, Epalrestat, glutathione, oxidative‐stress, Parkinson's disease

## Abstract

**Background:**

Parkinson's disease (PD) is a progressive neurodegenerative disorder affecting a large number of elderly people worldwide. The current therapies for PD are symptom‐based; they do not provide a cure but improve the quality of life. Muscular dysfunction is the hallmark clinical feature of PD and oxidative stress and inflammation play a critical role in its pathogenesis. Epalrestat is used for the treatment of diabetic neuropathy and is known to improve antioxidative defense mechanisms in the CNS. Therefore, in this study, we investigated the role of Epalrestat in the reserpine induced mouse model of PD.

**Method:**

We used Swiss Albino mice for the PD model and tested for akinesia/bradykinesia, muscular rigidity, palpebral ptosis, and tremor, as well as conducting swim and open field tests. Brain samples were used to determine oxidative stress parameters and infiltration of immune cells.

**Results:**

Epalrestat treatment significantly improved akinesia and bradykinesia, muscular dysfunctions, tremor level, and gait functions compared to the reserpine group. It also improved the latency in the swim test. Eplarestat significantly reduced lipid peroxidation and NO concentration in different brain tissues and increased the activity of antioxidative enzymes, glutathione, catalase, and superoxide dismutase. Furthermore, Epalrestat reduced neuroinflammation by reducing the number of infiltrating immune cells.

**Conclusion:**

Eplarestat improves muscular dysfunction in PD by reducing oxidative stress and inflammation.

## 1 INTRODUCTION

There are more than 385.4 million people in Asia aged 60 years or older, and there are more than 41.9 million people who are 80 years of age or older.^1^ Life expectancy is also increasing steadily in developing countries. Because of the large number of elderly people and the steady increase in the elderly population, chronic debilitating diseases that affect people over 60 years old are of major concern.^1^ Parkinson's disease (PD) is one such debilitating disease that afflicts the elderly. It is the second most common neurodegenerative disorder after Alzheimer's disease, affecting a significant number of people around the world. PD is a progressive neurodegenerative disorder and belongs to a group of conditions called movement disorders. The four cardinal motor symptoms of PD are tremor at rest, rigidity, bradykinesia or akinesia, and postural instability.^2^ This age‐related nervous system disorder affects 2%‐3% of people older than 65 years,^3^ and the number of people in this age group is estimated to double by 2030.^4^ The consequences of PD are a huge burden on society as a whole. In particular, in relation to our study, PD is prevalent and a significant societal burden in Bangladesh.^4^

The pathological features of PD are degeneration of the dopaminergic neurons in the substantia nigra pars compacta, and the presence of Lewy bodies.^5^, ^6^, ^7^ Dopamine concentration drops significantly in PD as a result of damage to dopaminergic neurons.^8^ Together, these effects result in abnormal neuronal firing leading to muscular dysfunction.

Therapy for PD is not specific but rather is highly symptom‐based and individualized. Furthermore, PD is currently incurable, so all available therapies are only able to improve the quality of life.^9^ This fact motivates the scientists around the world to look for optimum therapies for successful use in PD. The current treatment options are also not devoid of adverse effects, and therefore the search for better drugs with optimum effects is of prime importance.^9^, ^10^, ^11^

Inflammation is associated with a number of neurodegenerative disorders including Parkinson's disease.^12^, ^13^, ^14^ Therefore, the titration of inflammation is a strategy to manage PD. Oxidative stress mediated by free radicals has been reported to be involved in PD. Since the brain consumes a substantial amount of oxygen relative to other organs, it is highly vulnerable to oxidative stress.^15^, ^16^, ^17^ Because of its high lipid content, the brain is also highly susceptible to lipid peroxidation, which is considered to be the central feature of oxidative stress^18^ and responsible for the induction of damage to biomolecules such as DNA and proteins.^19^ These effects may eventually lead to inflammation, which further aggravates the functional outcome in PD.^14^, ^20^ A growing body of evidence has reported an increased level of oxidative stress and a decrease in the level of glutathione (GSH) in the brain of PD patients, resulting in damage to dopamine secreting neurons.^21^, ^22^, ^23^ Therefore, restoring the GSH level in the brain would be expected to protect the dopamine secreting neurons. Circulating neutrophils are known to express nNOS and release NO free radicals.^24^ NO free radicals contribute to poor outcomes in PD by inducing nitrosative stress.

Currently, Epalrestat is indicated for the management of diabetic neuropathy.^25^ It improves the antioxidative defense mechanism in the CNS.^26^ Through transcriptional regulation, Epalrestat increases intracellular GSH levels.^27^ Decreased GSH levels are common long‐term complications in patients who have diabetes. It has been found that Epalrestat improves morphological abnormalities of nerves in the rodent model of diabetes. One study explored the effect of Epalrestat in a cell culture model of oxidative stress, but little is known about its effects on the in vivo system, in particular the phenotypic outcome of its use in neurodegenerative disorders like PD.^28^ Therefore, in our current study, we evaluated the role of Epalrestat in a mouse model of PD.

## 2 MATERIALS AND METHODS

### 2.1 Animals

**Figure 1:**
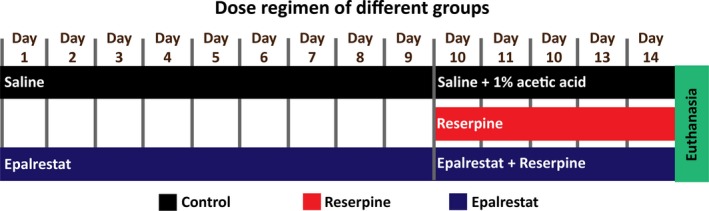
Dose regimen of the control, reserpine, and Epalrestat group. The control mice received saline (days 1‐14) and 1% acetic acid (days 10‐14) as a vehicle. The reserpine group received reserpine at a dose of 4 mg/kg subcutaneously (days 10‐14). The Epalrestat group received Epalrestat at a dose of 2 mg/kg orally (days 1‐9) along with reserpine at a dose of 4 mg/kg (days 10‐14)

Swiss Albino Mice (8‐12 weeks) were housed in standard housing conditions with access to unlimited food and water. Experiments were performed according to institutional guidelines and were approved by the North South University Animal Ethics Committee. All efforts were made to minimize the number of mice used and any pain or discomfort suffered by the animals. Three groups (control, reserpine, and Epalrestat) were established, consisting of eight mice in each group (four males and four females). The reserpine group was injected with reserpine (4 mg/kg) subcutaneously for five consecutive days according to existing protocols, with minor modifications.^29^, ^30^ Five milligrams (5 mg) reserpine was dissolved in 1 mL 1% acetic acid. This was then diluted ten times with distilled water to get a final concentration of 500 µg/mL. The treatment group received Epalrestat (2 mg/kg) by gavage twice daily for 14 days. During the last 5 days (days 10‐14), this group also received reserpine. The control group received saline for 14 days with 1% acetic acid from day 10 to day 14 (Figure 1: dose regimen illustration). The experimenters were blinded to the treatment group in all experiments. Analysis of the images for counting cells was performed blindly and counter checked by another blinded experimenter.

### 2.2 Behavioral tests

#### 2.2.1 Open field test

This experiment was performed according to existing protocols, with minor modifications.^30^ Mice were placed at the center of the open field and allowed to explore the apparatus for 10 minutes. Behavior was scored, and each trial was recorded with Logitech^TM^ 4mp webcam. The immobility duration was analyzed automatically using Smart^TM^ v3.0 video tracking software.^31^

#### 2.2.2 Akinesia/Bradykinesia

An akinesia test was performed to evaluate the movement impairment. In this test, the mouse was held by the tail so that it stood on its forelimbs and moved on its own. The number of steps taken with both forelimbs was recorded for 30 seconds.^32^ A bradykinesia experiment was done by placing the mouse forepaws on a horizontal wooden bar (7 mm in diameter), 40 mm above the tabletop. The time until the mouse removed both forepaws from the bar was recorded, with a maximum cut off time of 3 minutes.^30^, ^33^

#### 2.2.3 Tremor

The level of tremor (occurring at a frequency between 4 and 6 Hz) induced by reserpine was evaluated visually and scored as follows: 0, no tremor; 1, occasional isolated twitches; 2, moderate or intermittent tremor associated with short periods of calmness; and 3, pronounced continuous tremor.^30^, ^34^

#### 2.2.4 Palpebral ptosis

The ptosis of the eye was evaluated visually and scored according to the rating scale: 4, eyes completely closed; 2, half‐open eyes; and 0, wide‐open eyes; with 1 and 3 indicating intermediate values.^30^, ^35^

#### 2.2.5 Muscle rigidity

Mice were suspended by their forelimbs on a metal rod (25 mm in diameter) that was held approximately 200 mm above the surface. The number of steps taken with each forelimb when the mouse is pushed sideways over a distance of 500 mm was recorded.^32^, ^36^

#### 2.2.6 Forced swim test

The forced swim was performed in a stainless steel vessel (450 mm × 220 mm × 200 mm) filled with water to a depth of 100 mm.^37^ Mice were trained for two consecutive days, with training sessions consisting of 12 trials each 1 minute long, during which the mice had to find a platform located in one of the quadrants.^38^ After the training, the probe trial was performed during which the platform was removed. The mice were placed at a unique starting location opposite to that of the platform. During the probe trial, all movements were recorded and measured for 60 seconds.

#### 2.2.7 Gait alteration

In this test, both the paws and the limbs of the mice were stained with food‐grade color and the mice were placed on a clean white sheet of paper. The footprints were then used for the manual analysis of gait patterns,^39^ focusing on the following parameters: stride distance, sway distance, and stance distance.^40^

#### 2.2.8 Euthanasia

On day 5, mice were deeply anesthetized using ketamine. They were then transcardially infused using saline. Brains were removed quickly and dissected for the striatum, cerebral cortex, and cerebellum.

### 2.3 Biochemical assay

#### 2.3.1 Protein estimation

Protein concentration was estimated from brain and plasma samples according to the method described previously.^41^ Briefly, 20 µL samples were mixed with 20 µL of sodium hydroxide solution and heated at 100°C in a water bath for 10 minutes. After cooling to room temperature, 200 µL complex reagents were added and incubated for 10 minutes. Twenty microliters (20 µL) of Folin's reagent were then added and incubated for 60 minutes. The samples were then read at 750 nm.

#### 2.3.2 Malondialdehyde

The calculated amount of sample was mixed with 250 µL of trichloroacetic acid and centrifuged for 20 minutes. Five hundred microliters (500 µL) of supernatant was collected and mixed with 500 µL of thiobarbituric acid and incubated in the dark for 15 hours. The mixture was read at 630 nm. The concentration was determined using a standard curve.

#### 2.3.3 Superoxide dismutase

Autoxidation of epinephrine at pH 10.4 was spectrophotometrically measured. With this method, tissue supernatant was mixed with 0.02 ml epinephrine. After 5 minutes, the absorbance was measured at 480 nm. The activity of superoxide dismutase (SOD) was expressed as percentage activity of inhibition of autoxidation.^42^

#### 2.3.4 Catalase

An estimated number of samples were mixed with 50 µL of hydrogen peroxide. Changes in absorbance of the reaction solution at 405 nm were determined after 0, 30, 60, 120, 360, and 480 seconds. One unit of catalase activity was defined as an absorbance change of 0.01 as units/min.^43^

#### 2.3.5 Glutathione

The GSH concentration was measured according to the protocol described previously, with minor modifications.^44^ Twenty microliters (20 µL) of the sample were mixed with 80 µL of phosphate buffer and 20 µL of 5,5‐dithio‐bis (2‐nitrobenzoic acid) and incubated for 5 minutes. The absorbance was then read at 412 nm.

#### 2.3.6 Nitric oxide

The nitrite content was determined using Greiss reagent (0.1% N‐(1‐naphthyl) ethylenediamine dihydrochloride, 1% sulfanilamide, and 2.5% phosphoric acid) as described previously.^45^ Briefly, equal volumes of sample and Greiss reagent were mixed and incubated for 10 minutes at room temperature in the dark. The absorbance was determined at 450 nm. The concentration of nitric oxide (NO) was measured using a standard curve.

#### 2.3.7 Hematoxylin and eosin (H&E) staining

The brain samples were fixed in formalin and embedded in paraffin wax. The paraffin sections (5 μm thick) were deparaffinized in 100% xylene and kept in 100% ethyl alcohol for 10 minutes. Then the sections were rehydrated with graded concentrations (90%, 80%, 70%, and 50%; vol/vol in water) of ethyl alcohol for 5 minutes each. After washing with distilled water, the sections were stained with hematoxylin and eosin solution. The sections were washed using graded concentrations of ethyl alcohol (50%, 70%, 80%, 90%, and 100%; vol/vol in water) and finally immersed twice in 100% xylene. Then the slides were mounted and visualized under a light microscope (Axio Scope A1).

### 2.4 Statistical analysis

Values are presented as mean (±SEM). Groups were compared using either one‐way analysis of variance (ANOVA) and the Bonferroni multiple comparison test or two‐way repeated‐measures ANOVA followed by the Bonferroni multiple comparison test.

## 3 RESULTS

### 3.1 Epalrestat improves muscular dysfunctions in a mouse model of Parkinson's disease

**Figure 2:**
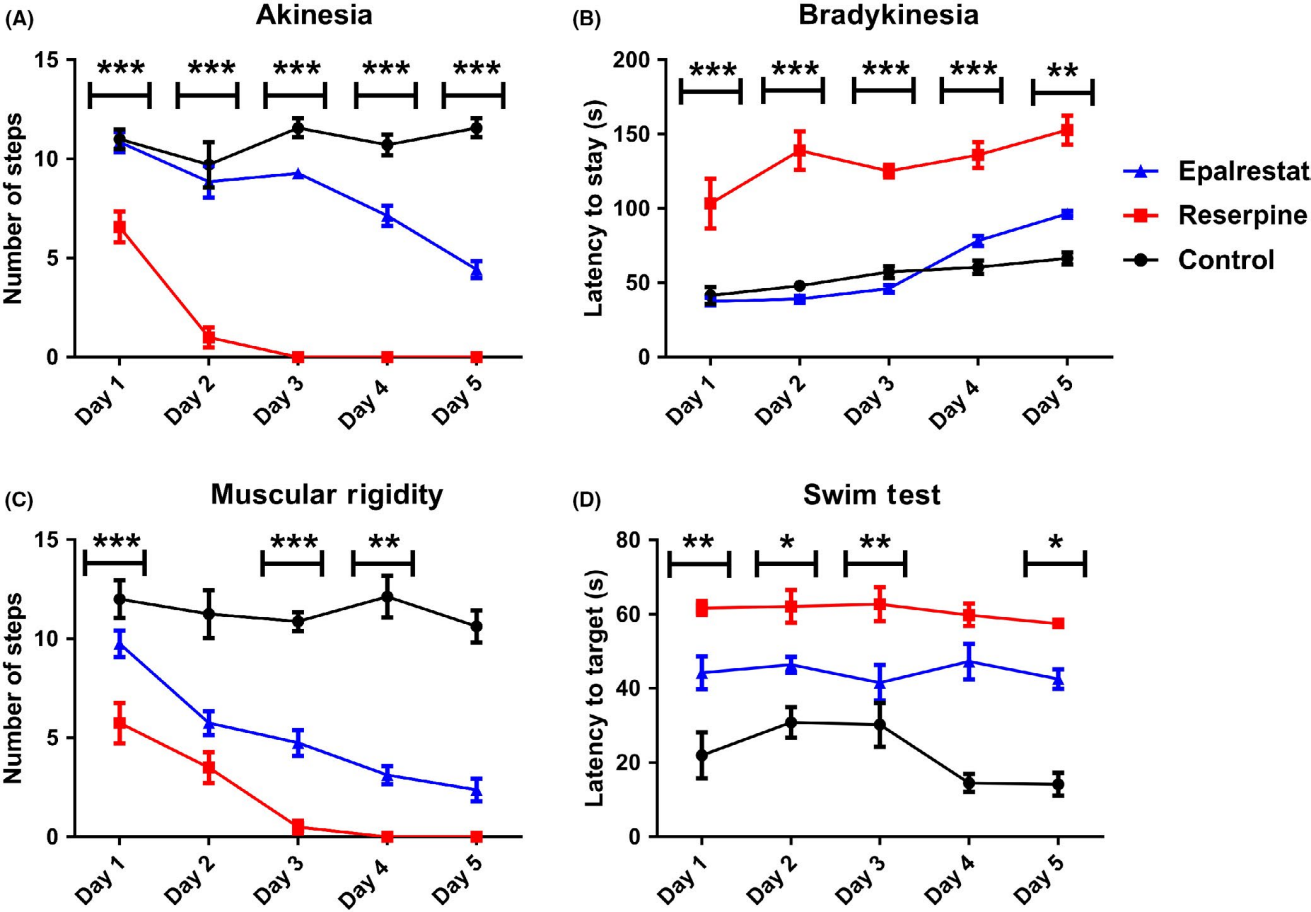
Epalrestat improves muscular dysfunctions in a mouse model of Parkinson's disease. Epalrestat treatment reduced the level of the following symptoms of muscular dysfunction compared to the reserpine group: A, akinesia (F (2,12) = 324.7, *P* < .0001); B, bradykinesia (*P* < .0001, F (2,14) = 127.9); C, muscular rigidity (*P* < .0001, F (2,14) = 126.8); D, latency to reach the platform in the swim test (*P* < .0001, F (2.75) = 118.6). Two‐way ANOVA followed by Bonferroni multiple comparison test. Asterisks (*) indicate a significant difference between Epalrestat and reserpine groups: *P < .05, **P < .01, ***P < .001. Values are mean (±SEM), n = 8

When mice were treated with reserpine for 5 days, they took substantially fewer numbers of steps with both forepaws when held by the tail compared to the control. However, treatment with Eplarestat resulted in an increased number of steps compared to the reserpine group (Figure 2A). Bradykinesia or slowness in movement is another common phenotype in Parkinson's disease and a hallmark feature of basal ganglia disorder.^2^ In our test for bradykinesia, reserpine treated mice took more time to remove their forepaws from the wooden bar compared to the control. Epalrestat treatment resulted in the normalization of this phenotype (Figure 2B). In line with this, muscular rigidity was also found to be reduced significantly with Epalrestat treatment (Figure 2C) compared to the reserpine group. In this test, mice treated with Epalrestat took a significantly greater number of steps in 1 minute (Figure 2C) compared to the reserpine group. The swim test is known to be associated with striatal dopamine function.^46^ The latency to reach the platform was longer in reserpine treated mice compared to the control. Epalrestat treatment improved this parameter significantly (Figure 2D). These results indicated that Epalrestat is a potential therapeutic agent for alleviation of Parkinson's disease‐like symptoms in mice.

### 3.2 Epalrestat prevents palpebral ptosis and tremor in a mouse model of Parkinson's disease

**Figure 3:**
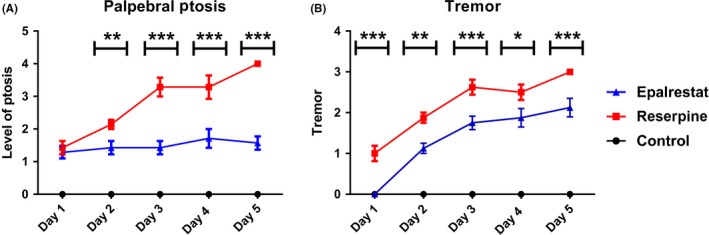
Epalrestat prevents palpebral ptosis and tremor in a mouse model of Parkinson's disease. A, treatment with Epalrestat resulted in a reduced level of ptosis compared to the reserpine group. Two‐way ANOVA, *P* < .0001, F (2,12) = 225.2, ***P < .001, **P < .01 (Bonferroni multiple comparison test; asterisks (*) indicate a significant difference between Epalrestat and reserpine groups). Values are mean (±SEM), n = 8. B, reserpine induced tremor level was also reduced significantly when treated with Epalrestat. Two way ANOVA, *P* = .0002, F (1,7) = 50.48, ***P < .001, **P < .01, ^*^ *P* < .05 (Bonferroni multiple comparison test; asterisks (*) indicate a significant difference between Epalrestat and reserpine groups).Values are mean (±SEM), n = 8

Palpebral ptosis refers to reduced eyelid elevation due to abnormal function of the levator palpebrae superioris, the primary muscle responsible for upper eyelid elevation.^47^ In Parkinson's disease, spontaneous eye blink rate was found to be reduced substantially.^48^, ^49^ In our study, we noticed a high level of ptosis in mice treated with reserpine. Epalrestat treatment resulted in a reduced level of ptosis compared to the reserpine group (Figure 3A). Reserpine induced tremor level was also significantly reduced in mice treated with Epalrestat (Figure 3B). These results suggest that levator muscle function can be improved with Epalrestat treatment.

### 3.3 Epalrestat prevents gait alteration in a mouse model of Parkinson's disease

**Figure 4:**
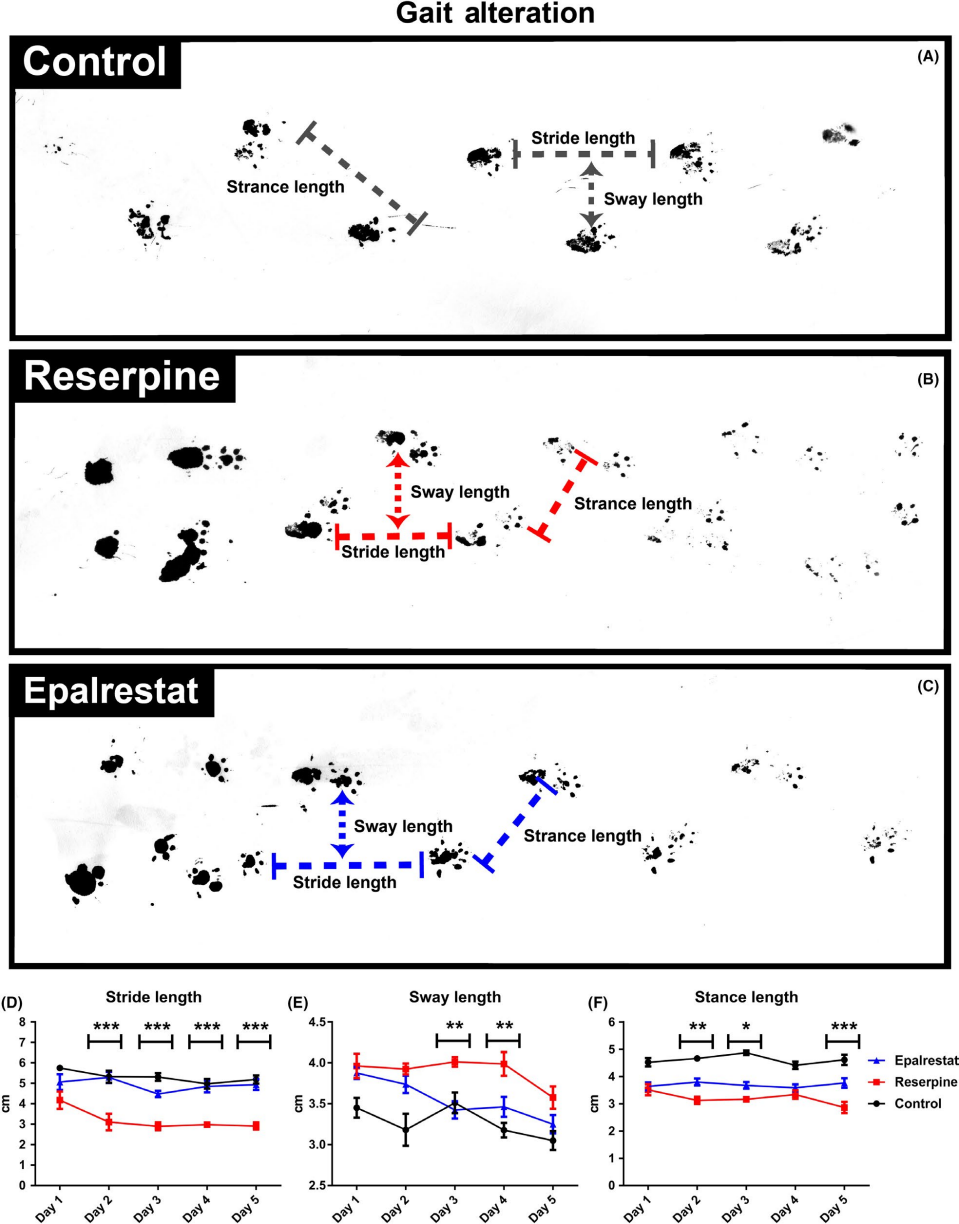
Epalrestat prevents gait alteration in a mouse model of Parkinson's disease. Figure shows the gait alternation in the control (A), reserpine (B) and Epalrestat (C) groups. Epalrestat treatment increased the stride length (D) and stance length (F). Two‐way ANOVA, *P* < .0001, F (2,14) = 75.83 (D), and F (2,14) = 82.49 (F), ***P < .001, **P < .01, *P < .05 (Bonferroni multiple comparison test; asterisks (*) indicate a significant difference between Epalrestat and reserpine groups). Values are mean (±SEM), n = 8. E, Epalrestat treatment also prevented altered sway length. Two‐way ANOVA, *P* < .0001, F (2,14) = 29.07, **P < .01 (Bonferroni multiple comparison tests; asterisks (*) indicate a significant difference between Epalrestat and reserpine groups). Values are mean (±SEM), n = 8

Postural instability and gait disturbances are common and late features of Parkinson's disease.^50^ We performed a gait abnormality test by analyzing the footprint of reserpine treated mice (Figure 4A‐C). Gait function in mice treated with reserpine was significantly affected, as evidenced by altered stride length (Figure 4D) and stance length (Figure 4F) in the footprint test. Epalrestat treatment increased the stride length and stance length, to lengths comparable to the control mice. Reserpine also decreased the sway length significantly compared to the control mice. Epalrestat treatment reversed this gait parameter to a normal level (Figure 4E). Thus Epalrestat may be a potential therapeutic agent for improving postural instability in Parkinson's disease.

### 3.4 Epalrestat enhances locomotor activity in a mouse model of Parkinson's disease

**Figure 5:**
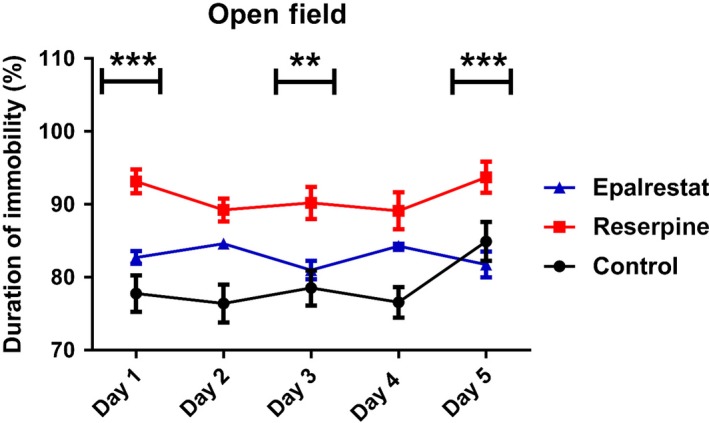
Epalrestat enhances locomotor activity in a mouse model of Parkinson's disease. Epalrestat treated mice were less immobile in the open field test compared to the reserpine group. Two‐way ANOVA, *P* < .0001, F (2,105) = 58.61, ***P < .001, **P < .01 (Bonferroni multiple comparison test; asterisks (*) indicate a significant difference between Epalrestat and reserpine groups). Values are mean (±SEM), n = 8

Locomotor activity was measured in mice using an open field test. We found that reserpine treated mice were significantly immobile compared to the control mice and Epalrestat treatment improved the mobility of the mice (Figure 5). Thus, Epalrestat improves locomotion in reserpine treated mice.

### 3.5 Epalrestat ameliorates reserpine induced oxidative stress in a mouse model of Parkinson's disease

**Figure 6:**
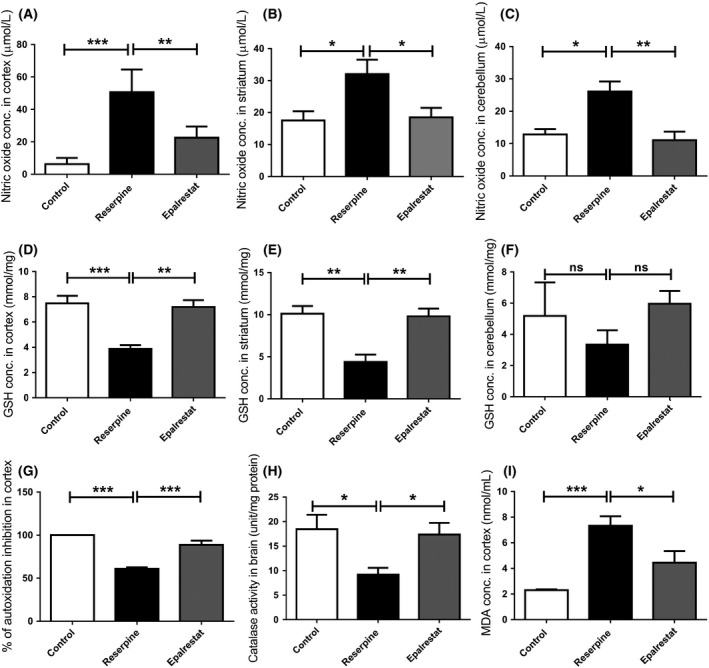
Effect of Epalrestat on oxidative stress‐induced brain damage. Epalrestat reduced the NO concentration in the following brian regions: A, cortex (*P* = .0003, F (2,9) = 23.58); B, striatum (*P* = .0210, F (2,14) = 5.158); C, cerebellum (*P* = .0043, F (2,9) = 10.57). ***P < .001, **P < .01, *P < .05. One‐way ANOVA followed by Bonferroni multiple comparison test. Values are mean (±SEM), n = 4‐7. Epalrestat also increased the brain glutathione concentration in: D, in cortex (*P* = .0004, F(2,10) = 19. 38); E, striatum (*P* = .0022, F (2,9) = 12.97); F, cerebellum (*P* = .4456, F (2,9) = 0.8856). ***P < .001, **P < .01, One‐way ANOVA followed by Bonferroni multiple comparison test. Values are mean (±SEM), n = 4‐7. G, Epalrestat treatment increased the superoxide dismutase medicated autoxidation inhibition significantly in cortex. One‐way ANOVA, *P* < .0001, F (2,10) = 32.58, ***P < .001(Bonferroni multiple comparison test). Values are mean (±SEM), n = 4‐7. H, Epalrestat treatment successful altered the activity of catalase in brain. One‐way ANOVA, *P* = .0146, F (2,19) = 5.323, *P < .05 (Bonferroni multiple comparison test). Values are mean (±SEM), n = 4‐7. I, Epalrestat reduced the malondialdehyde concentration significantly in cortex. *P* = .0004, F (2,15) = 13.88, ***P < .001, *P < .05. One‐way ANOVA (Bonferroni multiple comparison test). Values are mean (±SEM), n = 4‐7

The brain contains a high level of lipid and excitotoxic amino acids. It also has low levels of antioxidative enzymes. Hence it is more prone to oxidative stress than other organs.^51^ Oxidative stress is critically associated with neurodegenerative disorders including Parkinson's disease.^52^ NO and its breakdown products are key molecules that contribute substantially to eliciting and augmenting oxidative damage in neurodegenerative disorders.^53^, ^54^, ^55^ In our current study, reserpine significantly increased NO levels in different brain tissues (Figure 6A‐C), which were normalized upon Epalrestat treatment. Anti‐oxidative enzyme systems are present in the brain to scavenge the free radicals produced during oxidative stress. GSH plays a critical role in defense against reactive oxygen species (ROS). We noticed that the concentration of GSH decreased substantially in the cortex, striatum, and cerebellum in the reserpine treated mice. However, Epalrestat treatment restored GSH to normal levels, comparable to the control group (Figure 6D‐F). Likewise, SOD and catalase activities were increased in the brains of Epalrestat treated mice compared with reserpine treated mice (Figure 6G,H). In line with others,^56^ we noticed that reserpine induced lipid peroxidation in the cortex (Figure 6I) was significantly reduced upon Eplarestat treatment. These findings suggest that the neuroprotective effect of Epalrestat is linked with its antioxidant activity.

### 3.6 Epalrestat reduces the infiltration of immune cells in the brain of Parkinson's disease mouse model

**Figure 7:**
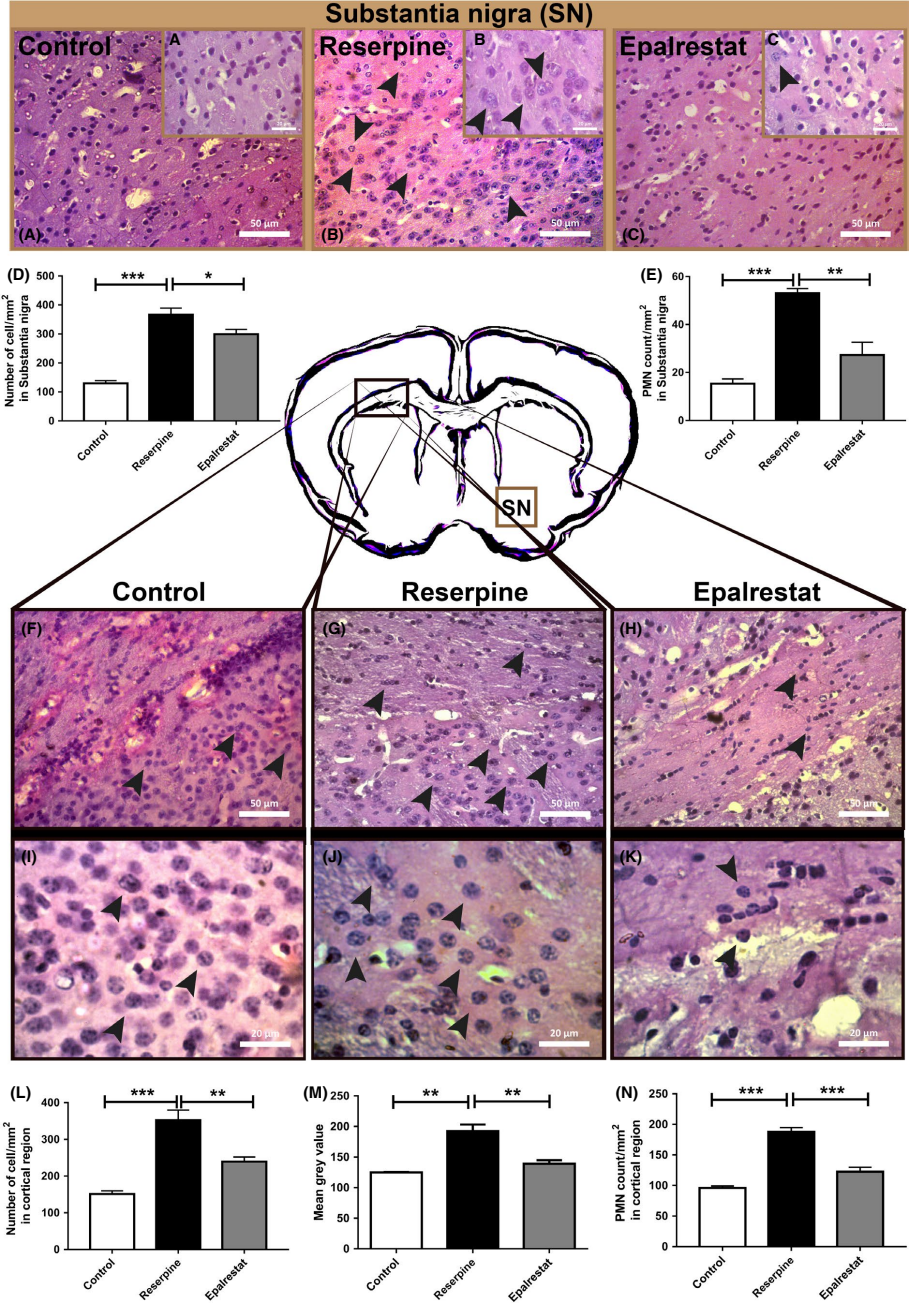
Epalrestat reduces the infiltration of immune cells in the brain of Parkinson's disease mouse model. Hematoxylin and Eosin staining of the immune cells of the substantial nigra region of the control (A), reserpine (B) and Epalrestat (C) group at 400× magnification and 1000× magnification in the insets at top right (a, b, c). The arrowheads indicate PMN cells. The staining revealed that Epalrestat treatment reduced the total number and number of PMNs in the substantia nigra (D and E, respectively). D, One‐way ANOVA, *P* < .0001, F (2,14) = 1.099, ***P < .001, *P < .05 (Bonferroni multiple comparison test). Values are mean (±SEM), n = 5‐6. E, One‐way ANOVA, *P* < .0001, F (2,13) = 2.04, ***P < .001, **P < .01 (Bonferroni multiple comparison test). Values are mean (±SEM), n = 4‐6. F‐H show the immune cells at 400× magnification in the cortical region of the control, reserpine and Epalrestat group, respectively. I‐K show the same cells at 1000× magnification. Treatment with Epalrestat also resulted in a reduced number of infiltrated cells in the cortical region of the brain (L and N). L, One‐way ANOVA, *P* < .0001, F (2,13) = 37.83, ***P < .001, **P < .01 (Bonferroni multiple comparison test). Values are mean (±SEM), n = 4. M, Mean gray value was also found to be reduced significantly with Epalrestat treatment. One‐way ANOVA, *P* = .0015, F (2,7) = 19.04, **P < .0021. Values are mean (±SEM), n = 3‐4. N, Epalrestat decreased the number of PMN cells in the brain of reserpine treated mice. One‐way ANOVA, *P* < .0001, F (2,9) = 55.73, ***P < .001 (Bonferroni multiple comparison test). Values are mean (±SEM), n = 4

Inflammation is a key player involved in the pathogenesis of Parkinson's disease and is thought to be associated with the progressive loss of dopaminergic neurons in the substantia nigra.^13^ We evaluated the cells in the substantia nigra (SN) and noticed that the number of cells was reduced in the Epalrestat group compared to the reserpine treated mice (Figure 7A‐D). When we evaluated the morphology of these cells, we noticed that a substantial proportion of these cell populations were polymorphonuclear (PMN) cells, having a characteristic feature of multi‐lobed nuclei (Figure 7B,C,J,K). Epalrestat also reduced the count of infiltrating PMN cells in the substantial nigral region (Figure 7A‐C,E). As in the SN region, reserpine treatment significantly increased the number of cells in the cortical region compared with the control (Figure 7F‐H) and this was normalized upon Epalrestat treatment (Figure 7F‐H,L). Treatment with Epalrestat also reduced the number of infiltrated PMN cells (Figure 7K,N). When we quantified these effects, the mean gray value was also found to be significantly reduced with Epalrestat treatment (Figure 7M). These results indicate that Epalrestat exerts an anti‐inflammatory effect in the mouse brain of Parkinson's disease.

## 4 DISCUSSION

Epalrestat ameliorates the behavioral deficit and muscular dysfunction in a mouse model of Parkinson's disease. Reserpine has long been used to induce Parkinson's‐like symptoms in rodents.^57^, ^58^, ^59^, ^60^ Reserpine depletes cellular monoamine content by inhibiting the vesicular transporter of monoamines (VMAT2) in the central nervous system.^61^, ^62^ In this study, we successfully induced the altered motor symptoms of Parkinson's disease upon injection of reserpine in Swiss Albino mice. These alterations in behavior associated with muscular dysfunction were reversed in most cases upon treatment with Epalrestat. Epalrestat also reduced the oxidative stress markers such as lipid peroxidation and NO, and inflammation associated with Parkinson's disease.

Oxidative stress plays a critical role in the pathogenesis of Parkinson's disease.^63^ A growing body of evidence suggests that degeneration of dopaminergic neurons in the substantia nigra pars compacta (SNpc) is associated with oxidative damage and mitochondrial dysfunction.^64^, ^65^, ^66^, ^67^ This association is further supported by the animal model of Parkinson's disease induced by chemicals such as reserpine.^68^, ^69^, ^70^, ^71^

Although comprising only 2% of body mass, the brain consumes about 20% of the oxygen supply in order to function properly. A substantial portion of this oxygen is converted to ROS. ROS can be produced in neurons and glial cells from many different sources such as iron, monoamine oxidase (MAO), NADPH oxidase (NOX), and other flavoenzymes.^72^ ROS generation in SNpc is also known to be a result of increased dopamine metabolism, low GSH, and high levels of iron and calcium.^66^ Apart from these effects, because of its high concentration of polyunsaturated fatty acids, the brain is highly susceptible to lipid peroxidation under oxidative stress conditions,^18^ affecting cell viability. Free iron in this context can react with lipid hydroperoxides generating alkoxyl radicals, which in turn perpetuate the lipid peroxidation.^73^ In our study, we also noticed a high level of lipid peroxidation in the brains of PD modeled mice (Figure 6I), which was normalized upon Epalrestat treatment.

Activation of enzymes such as nitric oxide synthase (NOS) or NADPH oxidases can also lead to the production of ROS.^74^ NOS, present in neurons (nNOS), endothelial cells (eNOS), and glia (iNOS), produces NO, which is found to in significant quantity in the extracellular fluid surrounding dopaminergic neurons and eventually impairs dopamine synthesis.^75^, ^76^, ^77^ Excess amounts of NO may lead to the production of the reactive nitrogen species (RNS) peroxynitrite (ONOO.−) by rapidly reacting with superoxide.^78^, ^79^ Peroxynitrite is known to induce DNA fragmentation and lipid peroxidation.^78^, ^79^ In line with this, we noticed an increased amount of NO in the brain of PD model mice, which was reduced significantly by Epalrestat treatment (Figure 6A‐C).

Defensive enzymes and antioxidant molecules that neutralize the ROS function are known to be produced in organisms as a mechanism for minimizing oxidative stress.^63^ GSH, catalase, and superoxide dismutase are antioxidant enzymes known to be present in the brain. Compromised GSH metabolism associated with the pathogenesis of Parkinson's disease has been described.^80^ In our study, we showed that reserpine treatment decreased the content of GSH in different brain tissues, and this was reversed by Epalrestat treatment (Figure 6D‐F). This finding is in line with a previous report by K Sato and colleagues showing that intracellular GSH levels could be increased in Schwann cells using Epalrestat via the transcription regulation of Nrf2.^27^ As well as increasing GSH concentration, we also noticed that Epalrestat increased the overall activity of superoxide dismutase and catalase in the brain of Parkinson's afflicted mice (Figure 6G,H).

Neuroinflammation is a common and prominent feature of PD and other neurodegenerative disorders.^13^ Activated microglia have been reported to mediate the neuroinflammation in PD.^81^ Activated microglial cells are an important source of superoxide and NO, which in turn contribute to oxidative and nitrosative stress in the brain.^63^ ROS are also known to activate the nuclear factor‐kappa‐B (NF‐κB), which in turn activates various pro‐inflammatory genes.^56^

The neutrophil is one of the critical immune cells involved in many inflammatory cascades linked to neurodegenerative diseases, including PD.^24^, ^82^, ^83^ It is capable of producing reactive oxygen species such as hypochlorous acid (HOCl), which converts dopamine into chlorodopamine, a toxicant to dopaminergic neuron.^84^ In our study, we noticed a significant increase in the infiltration of neutrophil cells in the brain of PD model mice, which was reversed by Epalrestat treatment. Morphological analysis of H&E stained brain sections revealed that a substantial portion of these infiltrated cells are PMN cells and Epalrestat treatment reduced the number of PMN cells in the cortical region of the PD brain (Figure 7). This finding is also in line with other reports showing that Epalrestat inhibits neutrophil‐endothelial cell adhesion and related surface expression of endothelial adhesion molecules.^85^, ^86^, ^87^ However, this finding should be confirmed by immunohistochemistry to further delineate the role of PMN cells in PD.

Endothelial adhesion is crucial for the infiltration of PMN cells. Intracellular adhesion molecule‐1(ICAM‐1), which is associated with regulation of adhesion and transcellular migration, is a key player in this context.^88^ Protein kinase C (PKC), on the other hand, appears to be necessary for the expression of ICAM‐1 on the endothelial cells.^89^ Multiple studies have reported the role of the aldose reductase pathway in activating PKC.^90^, ^91^ Since Epalrestat is an aldose reductase inhibitor, it might inhibit the expression of ICAM‐1 through inhibition of PKC activation and thereby reduce the number of infiltrating immune cells into the brain parenchyma, leading to reduced inflammation. Inhibition of PKC is also reported to be neuroprotective in an animal model of PD.^92^ NADPH oxidase present in phagocytes, monocytes, and other inflammatory cells serves as a potential source of ROS. ROS eventually activate PKC, resulting in activation of redox‐sensitive transcription factor NF‐ κB. This leads to the transcription of proinflammatory cytokines.

## 5 CONCLUSION

We conclude that Epalrestat is protective in the mouse model of PD, and this protection is associated with reduced oxidative stress and inflammation. Since Epalrestat is already in clinical practice for the treatment of diabetic neuropathy, the translation from bench to bedside in the treatment of PD would be easy upon further experimentation.

## CONFLICT OF INTEREST

None.

## AUTHOR CONTRIBUTIONS

MR, ZH, and RW planned and designed the study. MR, ZH, MA, AA, and RC wrote the manuscript. AP, AA, and MA performed oxidative stress assay. AA, OS, and RC performed H&E staining and analysis. FK, RA, HJ, RC, and AA performed the behavioral study. MR, RW, and ZH critically reviewed the manuscript and formulated the discussion part of the manuscript. MR and RC contributed equally to this manuscript.
